# Benzodiazepine Reduction and a Generalised Tonic-Clonic Seizure with Therapeutic Benefit in Catatonia associated with Autism Spectrum Disorder

**DOI:** 10.1097/YCT.0000000000000835

**Published:** 2022-03-01

**Authors:** Sarah Parry, Ali Raheem, Elliot Williams, Jonathan Rogers

**Affiliations:** 1 South London and Maudsley NHS Foundation Trust, London, UK; 2 East London NHS Foundation Trust, London, UK; 3 Windsor Clinical and Home Care Services Group Ltd, Maidenhead, UK; 4 Division of Psychiatry, University College London, London, UK

**Keywords:** Catatonia, Autism spectrum disorder, Intellectual disability, Benzodiazepine withdrawal, Seizure

Dear Sir,

We describe improvement in catatonia following a seizure related to benzodiazepine withdrawal. A 21-year-old woman with a history of preterm birth, patent ductus arteriosus, neonatal intraventricular haemorrhage, chronic compensated hydrocephalus, pre-diabetes mellitus, polycystic ovarian syndrome, mild intellectual disability and autism spectrum disorder was referred to outpatient mental health services. Her only previous seizure had been age 15, whilst taking sertraline and quetiapine and she was not subsequently on anticonvulsants. She had previously been treated since the age of 13 for social withdrawal, poor sleep, reduced appetite and hearing voices following a suspected bullying incident on a school trip, which gradually deteriorated, characterised by poor occupational function, lack of motivation and mutism. Previous trials of sertraline, quetiapine, risperidone and aripiprazole were unsuccessful.

Following transition to adult mental health services at the age of 18, she was re-assessed and it was observed that she presented with features of catatonia, including mutism, staring, stereotypy, waxy flexibility and tachycardia with a heart rate ranging from 135-145/min during clinical reviews, meeting DSM-5 criteria for Catatonic disorder NOS. Using the Bush Francis Catatonia Rating Scale (BFCRS), she scored 7/14 on the screening instrument and 16/69 for severity. She was treated for catatonia in autism with oral lorazepam (off-label use) with the intention of using ECT if this was unsuccessful. As an outpatient, lorazepam was titrated slowly over 10 months to a total daily dose of 16mg in divided doses without any improvement, so a decision was made to restart sertraline at 50mg and to wean down the benzodiazepine dose by switching to diazepam due to its longer half-life. Lorazepam 16mg was converted to diazepam 50mg (off-label) and sertraline was increased to 100mg.

Three days after the switch from lorazepam to diazepam, the patient had a generalised tonic-clonic seizure at home lasting 7 minutes. She was transferred to the Emergency Department, where she had a further seizure. There was evidence of a mild inflammatory response with a white cell count of 12.5 x 10^9^/L, CRP of 28mg/L, platelets of 454 x 10^9^/L, and initial lactate of 8.0mmol/L, which normalised on repeat. No cause of seizure was identified, and no evidence of other symptoms of benzodiazepine withdrawal were noted. She had no further seizures after discharge.

Following the seizure, the dose of diazepam was held at 50mg for three weeks prior to restarting the reducing regimen. For the first 2 days following the seizures she was post-ictal and drowsy. However, by day 7 the patient’s presentation changed and she was noted to become substantially more communicative. She was described as chatty and asked questions about her diagnosis and her medication for the first time in years. In the next few months, as diazepam was gradually reduced, the improvement in symptoms was sustained. She continued to communicate more, both verbally and by using messages on her mobile. Due to COVID-19 restrictions, the further reduction in diazepam has been managed in the community, with telephone consultations only, hitherto uneventfully, so monitoring with the BFCRS has not been possible. She was reviewed by cardiology 3 months after the seizure due to persistent tachycardia. By this time, the tachycardia had improved to 102, which was thought to be due to iron deficiency anaemia. Her improvement does not seem to have been sustained in the long term.

To our knowledge, this is the first report of improvement of catatonia following a seizure in the context of benzodiazepine reduction. Benzodiazepine equivalences are not universally agreed, but 50mg diazepam may be a lower dose than 16mg lorazepam, ^1^ which could have been a factor, in addition to her underlying vulnerabilities. Autism spectrum disorder is associated with an increased propensity to seizures.^2^ Furthermore, the increase in sertraline from 50mg to 100mg may have been a precipitating factor, as her only previous seizure also occurred when she was treated with sertraline. Finally, given the mild inflammatory response noted in the Emergency Department, an infection may have been a contributing factor.

There are a number of possible triggers for the improvement in the patient’s symptoms.

Firstly, the high dose of lorazepam may have had a sedative effect, which improved following a dose reduction. This seems unlikely, given that the patient had these symptoms prior to lorazepam treatment and benzodiazepines do not tend to cause features such as waxy flexibility, stereotypy and tachycardia.

Secondly, sertraline may have treated an underlying anxiety or depressive illness. Again, this seems improbable as sertraline had previously had little effect and the dramatic improvement occurred within days of increasing the dose.

Thirdly, we suggest that the most likely reason for the patient’s improvement is that the seizure was therapeutic for the catatonic state. Benzodiazepine withdrawal is known to sometimes precipitate catatonia. ^3^ However, electroconvulsive therapy is a recommended and evidence-based treatment for catatonia. Prior to the use of ECT, pharmacological provocation of seizures using a variety of agents had been used as a treatment of catatonia. ^4^ It is therefore plausible that a seizure induced by withdrawal of a medication might be equally effective.

Treatment of catatonia in autism is supported only by case reports and case series, which suggest that benzodiazepines and ECT should be used. ^5^ In this case, given that lorazepam was unsuccessful, ECT would have been an appropriate treatment and could have been considered given that her hydrocephalus was stable.

This report supports the notion that ECT should be considered early as a potential therapeutic modality in catatonia in autism even when there is no immediate risk to life. It also provides a caution about gradual titration of benzodiazepines in catatonia, as it can cause tolerance and may risk withdrawal seizures, especially in individuals with risk factors for seizures. Finally, this case illustrates a complex relationship between seizures and catatonia, since catatonia can be a manifestation of non-convulsive status epilepticus.

A number of features of this case may limit the applicability to many patients with catatonia and autism, given her complex medical history. However, it illustrates an important paradoxical response to a reduction in benzodiazepine dose.
